# Disc Injury and Spine Loads in Low-to-Moderate-Severity Frontal Impacts

**DOI:** 10.1007/s10439-025-03808-w

**Published:** 2025-07-19

**Authors:** Richard Kent, Jason Forman

**Affiliations:** 1 Center for Applied Biomechanics, University of Virginia, Charlottesville, VA USA; 2 Orthopaedic Surgery, University of Virginia, Charlottesville, VA USA; 3 Mechanical and Aerospace Engineering, University of Virginia, 122 Engineer’s Way, Charlottesville, VA 22903 USA

**Keywords:** Frontal impact, Spine loads, Spine injury, Disc injury

## Abstract

**Purpose:**

Determine the cervical and lumbar spine forces and moments generated in belted occupants in frontal impacts up to 40-km/h change in velocity (${\Delta }V$) and assess their potential to cause spinal disc injury.

**Methods:**

Loads experienced by anthropomorphic test devices, human volunteers, and human cadavers were measured in 282 impact tests. Functions were developed to describe the expected loads as functions of ${\Delta }V$ for small females, mid-size males, and large males. Loads were contextualized by comparison to injury assessment reference values, manual lifting standards, the compressive loads that have caused spinal disc injuries in vitro, and the compressive spinal loads that occur in other situations.

**Results:**

The functions confirm that the spinal loads are well below any established injury assessment reference value. Compressive loads are within the range of loads voluntarily tolerated during daily activities and well below the loads that have caused disc injuries in biomechanical studies.

**Conclusion:**

This study confirms the lack of a biomechanical foundation to assert specific causation when a belted occupant presents with isolated disc herniations, bulges, or extrusions through the annulus following a frontal impact under 40-km/h ${\Delta }V$.

**Supplementary Information:**

The online version contains supplementary material available at 10.1007/s10439-025-03808-w.

## Introduction

Acute injuries to the spinal discs are exceedingly rare in motor vehicle collisions (MVC), occurring at a rate of approximately 1 per 1 million occupants exposed [1]. Degenerative disc disease, on the other hand, is common in the adult population and can cause bulges of the annulus fibrosis and even extrusions of nucleus through the annulus regardless of exposure to a MVC [2–9]. Patients and clinicians may attribute such pathology to an MVC and identify the pathology as the cause of post-collision symptoms [10–24].

When a dispute arises regarding causation of disc pathology, the circumstances of the MVC and its potential to generate loading in the spine can inform a resolution [2, 25–27], especially in low-speed collisions where injury outcomes may be defined largely or wholly by self-reported symptoms, and the pre-collision state of the pathological disc is unknown. Unfortunately, there are important gaps in the literature addressing spinal loads in low-speed collisions. Cervical spine loads in low-speed rear impacts have been studied extensively and, in fact, are used to provide consumer information ratings of contemporary vehicle seats and head restraints in 16-km/h rear impacts [28]. The cervical spine loads generated in other low-speed collision modes are less well documented, however, and lumbar spine loading in low-speed collisions is scarcely reported, regardless of collision mode [14, 29–31].

The purpose of this study, therefore, is to quantify the loads generated in the cervical and lumbar spines of adults in a common low-to-moderate-severity impact mode: frontal impacts below 40-km/h change in velocity (${\Delta }V$) and assess their potential to cause spinal disc injury.

## Methods

All data sources are detailed in the supplemental material. Test data were collected from sled or vehicle tests involving belted human volunteers, human cadavers, or anthropomorphic test devices (ATDs) outfitted with spinal load cells. The data sources were the published literature, the National Highway Traffic Safety Administration’s Biomechanics Test Database and Vehicle Crash Test Database, and the sled test archive at the University of Virginia Center for Applied Biomechanics [29–41]. Impacts with a ${\Delta }V$ less than or equal to 40 km/h and a principal direction of force (PDOF) 0f 0° were included. Unbelted occupants were excluded.

Cervical spine loads in the human volunteers and cadavers were determined from measured head kinematics using inverse dynamics methods, as described in the original publications. In general, tests involving human cadavers [32, 34, 35, 40] utilized accelerometer arrays or other instruments mounted rigidly to the head, typically with screws, potting materials, or both, to measure 6-degree-of-freedom kinematics at a point on the skull. The head’s geometry was then used to transform the kinematics to the head’s center of mass. The total mass and moments of inertia of the head were then estimated or measured directly, and Newton’s laws were used to calculate the forces and moments in the region of the occipital condyles required to cause the measured head kinematics. Neck loads were estimated similarly in the tests involving human volunteers [34, 36–38, 40], except head kinematics were measured using instruments attached less invasively to the head (e.g., with straps or bite blocks). The ATDs used were the 5th percentile female (AF5) Hybrid III and THOR dummies; the 50th percentile male (AM50) Hybrid III and THOR dummies; and the 95th percentile (AM95) Hybrid III dummy. Those ATDs can be outfitted with an upper-neck load cell that measures the components of the force and moment vectors. They also can be outfitted with a lower-neck load cell, and the THOR family can be outfitted with a load cell at the occipital condyle (OC). For this study, force and moment components were taken from the upper-neck load cell in all dummy tests, unless that signal was unavailable, in which case force components were taken from the lower-neck load cell or the OC load cell. Moment components were not included if they were unavailable from the upper-neck load cell. These loads were termed the “cervical spine loads.”

The Hybrid III ATDs can be outfitted with a lumbar spine load cell that measures the three components of the force and moment vectors, and the THOR family can be outfitted with a similar load cell nominally located at the 12th thoracic vertebra. These loads were termed the “lower spine loads.” No lower spine loads were available from the human volunteer or cadaver tests.

The tested ${\Delta }V$ was either extracted from the source documentation or calculated by integrating accelerometers mounted on the vehicle or sled. Peak values of the following force and moment components in the cervical and lower spine were compiled from either figures or tables in the source papers or reports, or raw data signals filtered per SAE J211 [42]: tension ($+ F^{z}$), compression ($- F^{z}$), the magnitude of anterior–posterior (A-P) shear ($\left| {F^{x} } \right|$), extension moment ($- M^{y}$), flexion moment ($+ M^{y}$), and the magnitude of lateral flexion moment ($\left| {M^{x} } \right|$). All data were debiased.

Data were scaled to represent the AF5, AM50, and AM95 ATD sizes [43] using the concept of similarity in dynamical systems [44, 45]. Several implementations of these fundamental scaling methods were considered [46–52]. As this study considers only adults, a constant-modulus, constant-stress, constant-density approach was chosen [45, 47, 53]. The whole-body mass ($m_{subj}$) and overall stature ($L_{subj}$) of each test subject were extracted from the source documentation, and the target masses ($m_{target}$) and heights ($L_{target}$) were taken from Reed and Rupp [43] for the AF5, AM50, and AM95 ATDs. A characteristic length ratio, $\lambda_{L}$, was defined for each test subject as $L_{target}$/$L_{subj}$, as was a characteristic mass ratio as $\lambda_{m} = m_{target}$/$m_{subj}$. The assumption of constant density yields a relationship between those ratios: $\lambda_{m} = \lambda_{L}^{3}$. The constant-stress assumption, combined with Newton’s 2nd law of motion, yields relationships between $\lambda_{L}$, $\lambda_{m}$, and the ratios of forces, $\lambda_{F} = F_{target}$/$F_{subj}$ and moments, $\lambda_{M} = M_{target}$/$M_{subj}$:

$$\lambda_{F} = \lambda_{m}^{{{\raise0.7ex\hbox{$2$} \!\mathord{\left/ {\vphantom {2 3}}\right.\kern-0pt} \!\lower0.7ex\hbox{$3$}}}} = \lambda_{L}^{2}$$ and $$\lambda_{M} = \lambda_{m} = \lambda_{L}^{3}$$.

The *i*th component of the force, $F_{subj}^{i}$, and moment, $M_{subj}^{i}$, vectors acting on the spines of the test subjects could therefore be scaled to the target occupant sizes using either mass scaling or length scaling as

$$F_{target}^{i} = \lambda_{L}^{2} F_{subj}^{i}$$ or $$F_{target}^{i} = \lambda_{m}^{{{\raise0.7ex\hbox{$2$} \!\mathord{\left/ {\vphantom {2 3}}\right.\kern-0pt} \!\lower0.7ex\hbox{$3$}}}} F_{subj}^{i}$$

and

$$M_{target}^{i} = \lambda_{L}^{3} M_{subj}^{i}$$ or $$M_{target}^{i} = \lambda_{m} M_{subj}^{i},$$

where $$i = x, y, z.$$

Both length and mass scaling were applied. The results did not differ meaningfully, so the length-scaled results are reported here. Both raw data and scaled data are reported.

Mathematical models were developed to describe the spinal loads as functions of ${\Delta }V$ for the unscaled data and for each occupant size. The expected load value was modeled using a simple two-parameter mathematical form to impose zero-valued load at zero ${\Delta }V$ and to reflect the observed and expected general monotonicity between load, expressed in units of N or Nm, and ${\Delta }V$ expressed in units of km/h:

1 $$Expected Load = A \cdot {\Delta }V^{B}.$$

A generalized reduced gradient nonlinear optimization method [54–56] was used to solve simultaneously for the parameters A and B that maximized the model fit to each set of scaled experimental data. The objective function to minimize was the sum of the squared error between the measured load and the model-predicted load over the entire ${\Delta }V$ range of 0 km/h to 40 km/h.

The range in the loads measured in these tests was described using 3rd-order polynomials to characterize the extreme upper and lower values of load measured in any test over the ${\Delta }V$ range included in this study. Four control points were defined for each polynomial: zero value at zero ${\Delta }V$, maximum or minimum value at or near 40-km/h ${\Delta }V$, and maximum or minimum value at two intermediate ${\Delta }V$ values. Those four control points define the three coefficients of each polynomial:

2 $$Extreme Load = C \cdot {\Delta }V + D \cdot {\Delta }V^{2} + E \cdot {\Delta }V^{3}.$$

This method allowed for a convenient method to define a region that is guaranteed to bound any load value measured in any test included in the study.

Finally, the compressive spinal loads in these frontal impacts were contextualized by comparison to injury assessment reference values (IARVs), manual lifting standards, and the compressive spinal loads that occur in other situations.

## Results

### Spinal Loads in Low-to-Moderate-Severity Frontal Impacts

As summarized in the Supplemental Material, 282 tests met the study inclusion criteria, 178 of which involved an ATD, 50 a human volunteer, and 48 a human cadaver. Occupants ranged in stature from 1513 to 1910 mm (mean 1730 mm) and in mass from 46.7 to 102.5 kg (mean 74.8 kg). ${\Delta }V$ ranged from 4.4 to 40 km/h (mean 25.1 km/h). An airbag provided supplemental restraint to 23 of the test subjects. No spinal disc injury was reported in any of the human volunteers or cadavers included in this study.

**Fig. 1:**
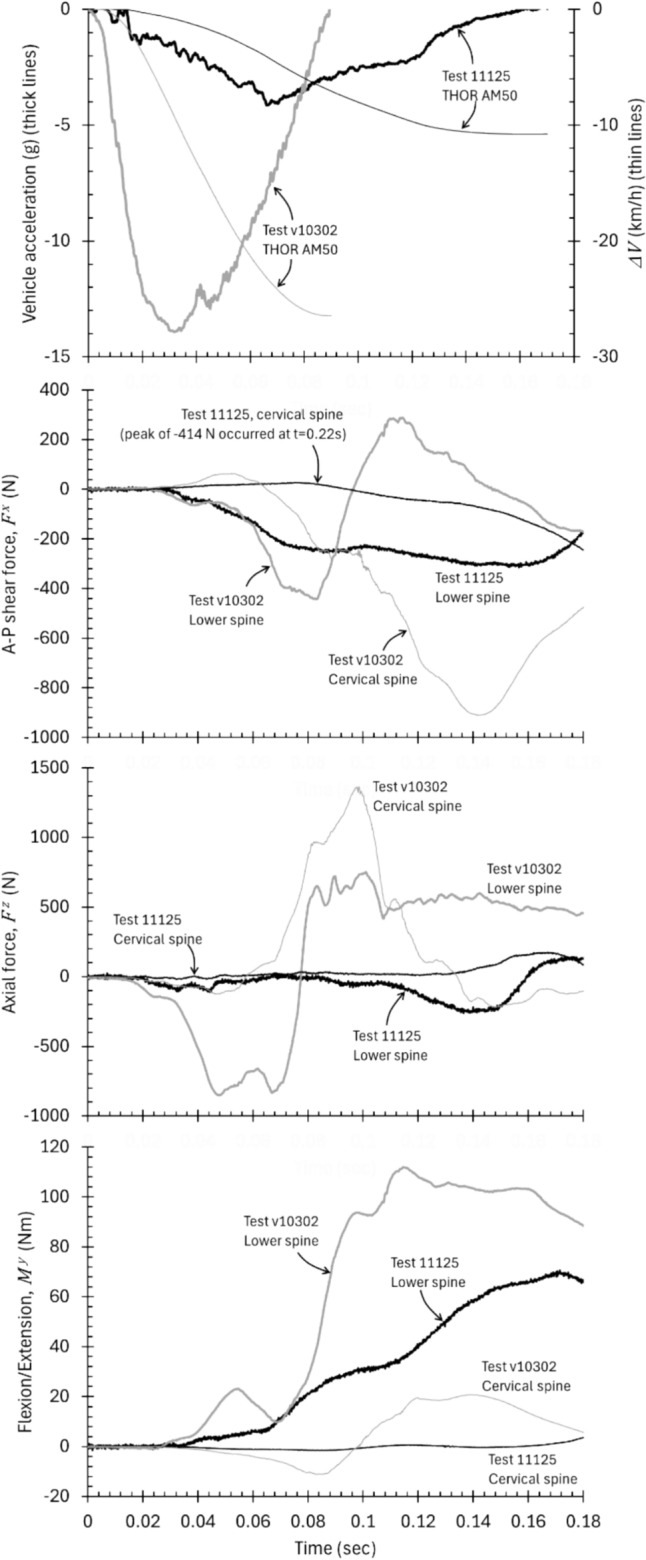
Example data traces from which peak values were extracted in tests toward the upper and lower ends of the severity range considered in this study

Example data traces reflect the temporal progression of spinal loading in two tests toward the upper and lower ends of the severity range considered in this study, and the peak values extracted from those tests for analysis (Figure 1) [41]. Test 11125 involved a universal vehicle buck with a ${\Delta }V$ of 10.8 km/h and an impact duration of 170 ms, while test v10302 involved a 2016 Chevrolet Malibu vehicle buck with a ${\Delta }V$ of 26.5 km/h and an impact duration of 90 ms. Both tests used a THOR AM50 ATD. In the higher-speed test, A-P shear in the lower spine peaked at − 442 N at 83 ms, while it peaked at − 910 N at 143 ms in the cervical spine. In the lower-speed test, A-P shear in the lower spine peaked at − 310 N at 157 ms, while the cervical spine did not reach peak A-P shear until rebound at 220 ms. The axial forces in the higher-speed test exhibited a compressive phase in the lower spine, reaching − 851 N at 48 ms, followed by a tensile phase that peaked at 751 N at 101 ms. The cervical spine experienced very little compression but reached a tensile peak of 1365 N at 98 ms. In the lower-speed test, the lower spine experienced negligible tension and reached an axial compression level of 252 N at 140 ms, while the cervical spine experienced negligible compression and reached a peak tension of 174 N at 165 ms. In both tests, the lower spine experienced negligible extension moment. Lower spine flexion moment peaked at 112 Nm at 115 ms in the higher-speed test and 70 Nm at 171 ms in the lower-speed test. The cervical spine experienced bimodal low-magnitude flexion-extension moment in the higher-speed test and very low-magnitude flexion moment in the lower-speed test.

**Fig. 2:**
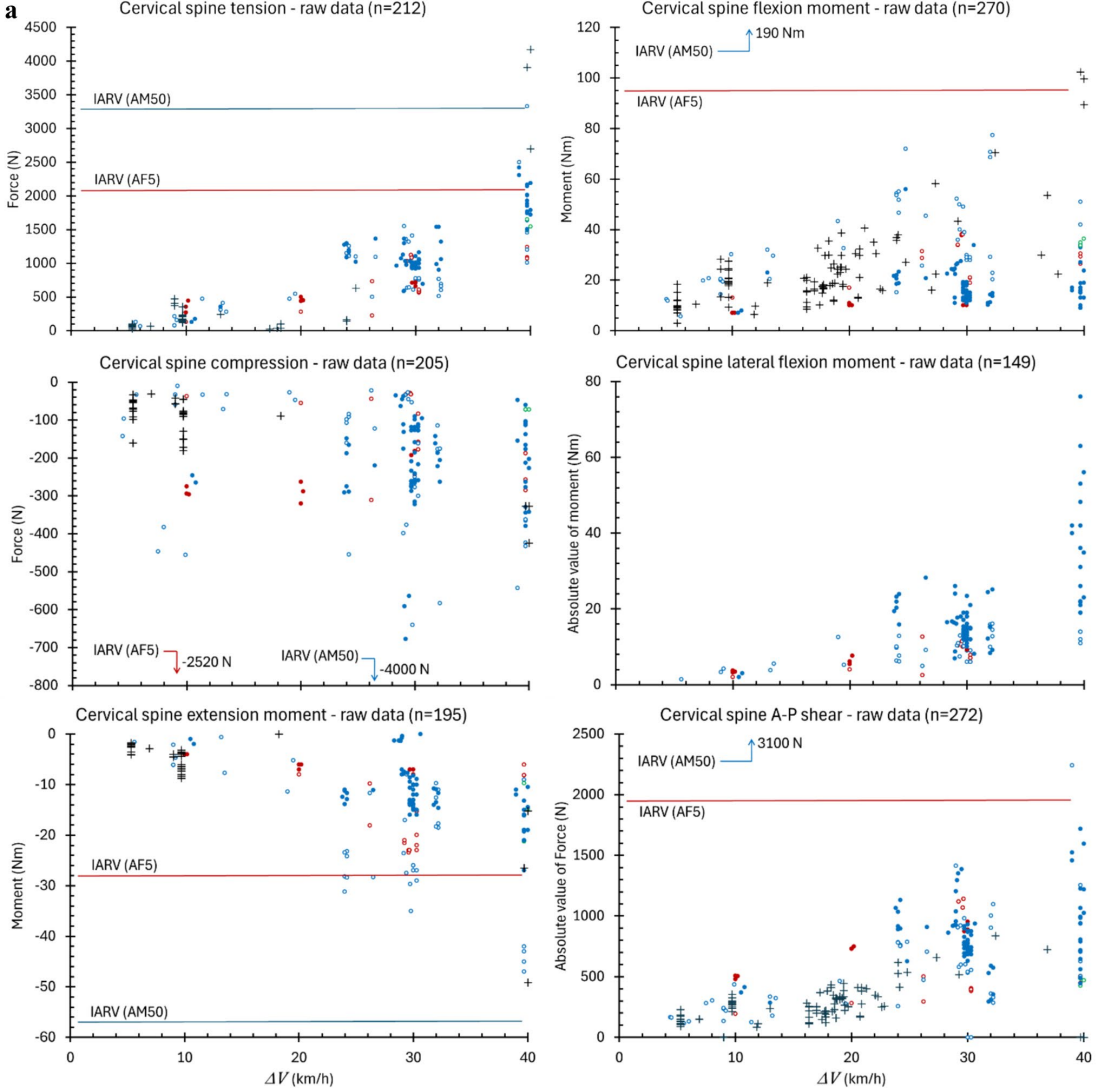

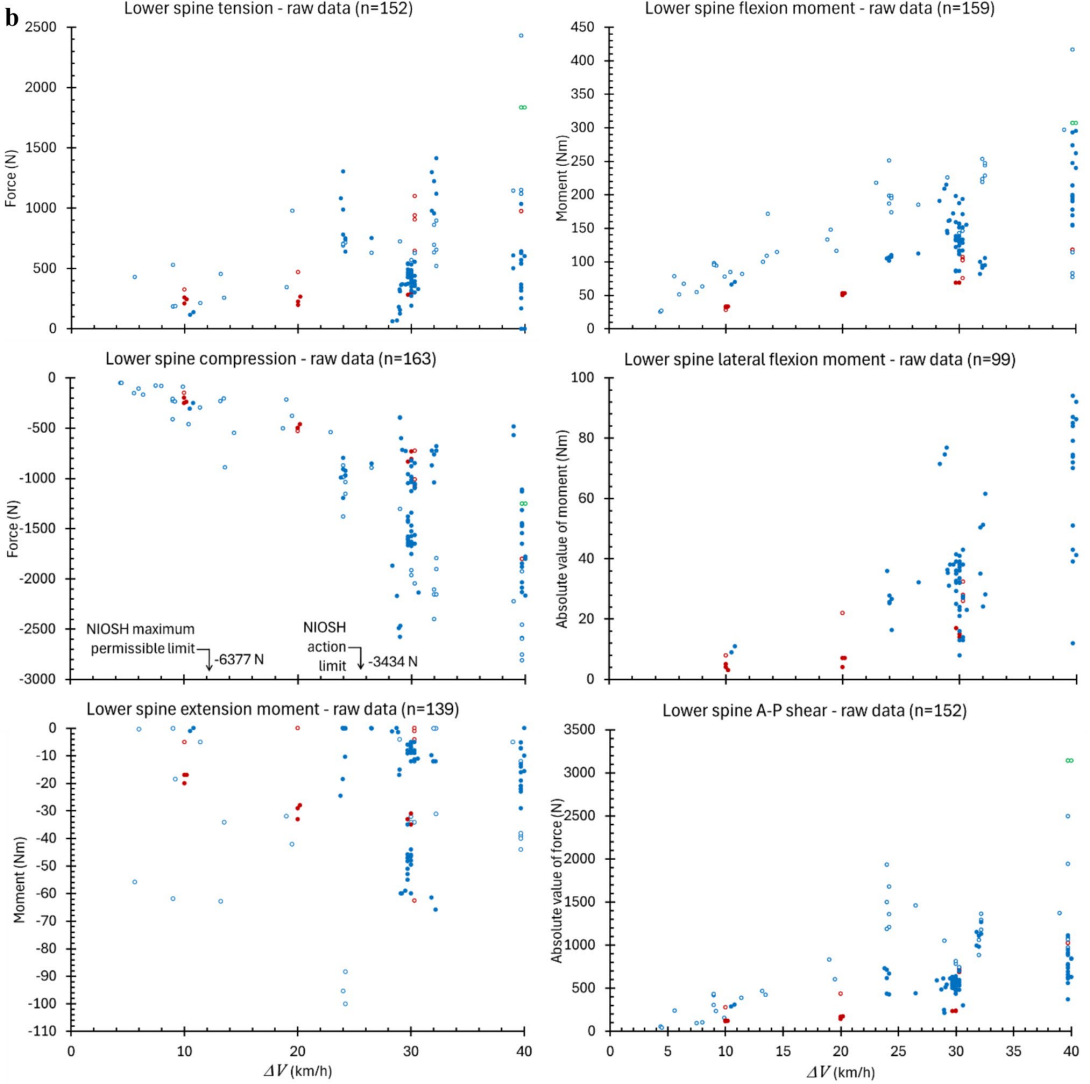
**a** Peak values of cervical spine loads in all tests meeting the inclusion criteria compared to IARV (tolerance values) from NHTSA [57]. Filled circles are data from the Hybrid III ATD family and empty circles from THOR. Red is AF5, blue is AM50, and green is AM95. Crosses are data from human volunteers or cadavers. **b** Peak values of lower spine loads in all tests meeting the inclusion criteria compared to NIOSH safe handling limits for compression [58, 59]. Filled circles are data from the Hybrid III ATD family and empty circles from THOR. Red is AF5, blue is AM50, and green is AM95

Cervical spine flexion moment was the most frequently measured load (270 of 282 tests) while lower spine lateral flexion moment was the least (99 tests). As expected, peak spinal loads were found generally to increase with increasing ${\Delta }V$, though substantial variance was observed across the ${\Delta }V$ range (Figure 2). This variance reflects differences in test conditions, including occupants, seats, seatbelts, deceleration profiles, and occupant engagement with vehicle interior and other structures. Overall, peak cervical spine tension was nominally a factor of two or more greater than spinal compression, reaching a maximum of 4170 N in one 40-km/h test. No occupant experienced more than 677 N of compressive force in the cervical spine. Cervical spine flexion moment was likewise a factor of approximately two greater than extension moment. No occupant experienced a cervical extension moment greater than 49.2 Nm or flexion moment greater than 102.4 Nm. No occupant experienced a lateral flexion moment greater than 76 Nm in the cervical spine. The greatest A-P shear measured in any occupant’s cervical spine was 2243 N.

In contrast to the cervical spine, the lower spine experienced slightly greater compressive force than tensile. Peak compression averaged 1826 N in the 48 occupants tested at 40-km/h ${\Delta }V$, while peak tension averaged 808.4 N for those occupants. The greatest A-P shear measured in any occupant’s lower spine was 3144 N.

**Fig. 3:**
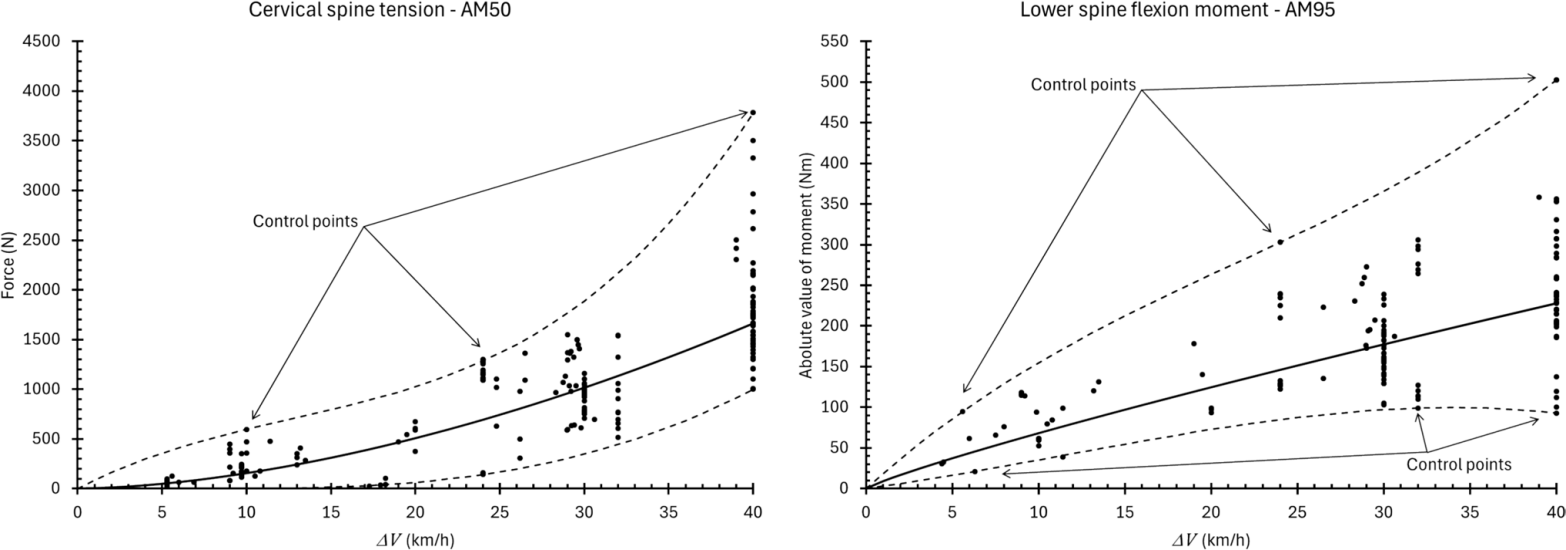
Examples of models fit to data. Expected value (Eq. (1)) shown in solid lines. Extreme upper and lower ranges (Eq.(2)) shown in dashed lines with control points identified. Model fits to all data are reported in the supplemental materials

Equations (1) and (2) describe the expected and extreme values of spinal loads measured in these tests (see Figure 3, Tables 1 and 2, Supplemental Material).

**Table 1:** Model coefficients for cervical spine loads (see Eqs. (1) and (2))

	Tension $+ F^{z}$	Compression $- F^{z}$	A-P Shear $\left| {F^{x} } \right|$	Flexion $+ M^{y}$	Extension $- M^{y}$	Lateral Flexion $\left| {M^{x} } \right|$
	Unscaled data					
*A*	2.885	− 18.054	20.131	8.265	− 0.455	0.036
*B*	1.723	0.675	1.037	0.280	0.999	1.743
*C*_upper_	73.031	− 77.282	61.569	3.623	− 0.373	0.265
*D*_upper_	− 3.137	2.532	− 1.426	− 0.033	− 0.064	0.015
*E*_upper_	0.09793	− 0.02327	0.03389	0.000162	0.00107	0.000652
*C*_lower_	8.988	− 1.566	9.258	0.572	− 0.408	0.114
*D*_lower_	− 1.019	0.064	− 1.337	− 0.004	0.036	− 0.010
*E*_lower_	0.03548	− 0.00140	0.03429	− 0.000135	− 0.00073	0.00034
	AF5					
*A*	2.654	− 14.197	15.045	5.836	− 0.394	0.067
*B*	1.666	0.672	1.038	0.268	0.915	1.453
*C*_upper_	60.956	− 57.570	68.373	1.970	− 0.321	0.104
*D*_upper_	− 2.288	1.871	− 2.152	0.002	− 0.032	0.026
*E*_upper_	0.06323	− 0.01695	0.03846	− 0.00025	0.00042	0.000048
*C*_lower_	14.841	− 1.162	6.099	0.405	− 0.276	0.275
*D*_lower_	− 1.411	0.047	− 0.881	− 0.009	0.028	− 0.019
*E*_lower_	0.03766	− 0.00104	0.02259	0.000056	− 0.00061	0.00040
	AM50					
*A*	2.971	− 17.194	20.151	9.039	− 0.608	0.064
*B*	1.715	0.702	1.038	0.268	0.916	1.595
*C*_upper_	87.679	− 77.282	91.582	3.545	− 0.509	0.063
*D*_upper_	− 3.819	2.532	− 2.882	− 0.029	− 0.048	0.047
*E*_upper_	0.09977	− 0.02327	0.05151	0.00011	0.00064	− 0.000025
*C*_lower_	1.537	− 6.420	8.170	0.644	− 0.415	0.423
*D*_lower_	− 0.424	0.373	− 1.180	− 0.015	0.040	− 0.029
*E*_lower_	0.02526	− 0.00615	0.03026	0.00011	− 0.00087	0.00062
	AM95					
*A*	3.346	− 19.974	23.005	10.973	− 0.738	0.082
*B*	1.716	0.694	1.034	0.266	0.915	1.578
*C*_upper_	92.520	− 87.407	103.682	4.271	− 0.468	0.194
*D*_upper_	− 3.473	2.841	− 3.254	− 0.034	− 0.079	0.049
*E*_upper_	0.09597	− 0.02576	0.05813	0.00013	0.00138	0.00009
*C*_lower_	10.687	− 1.764	9.258	0.773	− 0.501	0.509
*D*_lower_	− 1.247	0.072	− 1.337	− 0.018	0.049	− 0.034
*E*_lower_	0.04220	− 0.00157	0.03429	0.00013	− 0.00105	0.00075

**Table 2:** Model coefficients for lower spine loads (see Eqs. (1) and (2))

	Tension $+ F^{z}$	Compression $- F^{z}$	A-P Shear $\left| {F^{x} } \right|$	Flexion $+ M^{y}$	Extension $- M^{y}$	Lateral Flexion $\left| {M^{x} } \right|$
	Unscaled data					
*A*	36.692	− 8.377	8.334	7.228	− 0.00124	0.009
*B*	0.758	1.456	1.272	0.878	2.614	2.438
*C*_upper_	89.526	3.314	22.462	15.819	− 12.184	− 1.223
*D*_upper_	− 2.588	− 6.479	3.958	− 0.356	0.419	0.251
*E*_upper_	0.04672	0.11381	− 0.06388	0.00551	− 0.00356	− 0.00405
*C*_lower_	19.193	9.418	3.956	2.808	0.000	− 0.033
*D*_lower_	− 0.893	− 1.487	0.064	− 0.001	0.000	0.015
*E*_lower_	0.01034	0.02376	0.00174	− 0.00051	0.00000	− 0.00017
	AF5					
*A*	33.877	− 8.210	6.189	4.689	− 0.00123	0.009
*B*	0.697	1.390	1.274	0.885	2.496	2.317
*C*_upper_	66.855	4.899	3.797	7.304	− 7.596	− 0.320
*D*_upper_	− 1.933	− 4.982	4.079	− 0.036	0.218	0.134
*E*_upper_	0.03490	0.08706	− 0.07199	0.00054	− 0.00115	− 0.00220
*C*_lower_	14.656	7.027	2.947	1.699	0.000	0.363
*D*_lower_	− 0.687	− 1.110	0.048	0.020	0.000	− 0.013
*E*_lower_	0.00801	0.01774	0.00129	− 0.00078	0.00000	0.00021
	AM50					
*A*	39.012	− 12.807	6.573	10.017	− 0.00123	0.027
*B*	0.740	1.359	1.338	0.796	2.614	2.125
*C*_upper_	89.526	3.314	5.085	15.819	− 11.789	− 0.499
*D*_upper_	− 2.588	− 6.479	5.463	− 0.356	0.339	0.208
*E*_upper_	0.04672	0.11381	− 0.09642	0.00551	− 0.00180	− 0.00342
*C*_lower_	19.619	− 11.465	3.956	2.633	0.000	0.487
*D*_lower_	− 0.919	0.880	0.064	0.031	0.000	− 0.015
*E*_lower_	0.01071	− 0.02321	0.00174	− 0.00121	0.00000	0.00027
	AM95					
*A*	44.214	− 11.784	8.909	9.173	− 0.00124	0.010
*B*	0.740	1.415	1.288	0.871	2.664	2.447
*C*_upper_	101.46	3.815	22.898	19.088	− 14.221	− 0.591
*D*_upper_	− 2.933	− 7.346	5.049	− 0.429	0.409	0.250
*E*_upper_	0.05296	0.12902	− 0.09141	0.00665	− 0.00216	− 0.00411
*C*_lower_	20.501	10.662	4.480	2.898	0.000	0.162
*D*_lower_	− 0.937	− 1.685	0.073	0.088	0.000	0.006
*E*_lower_	0.01061	0.02692	0.00197	− 0.00255	0.00000	− 0.000037

### Loads That Have Caused Disc and Other Spinal Injuries

**Fig. 4:**
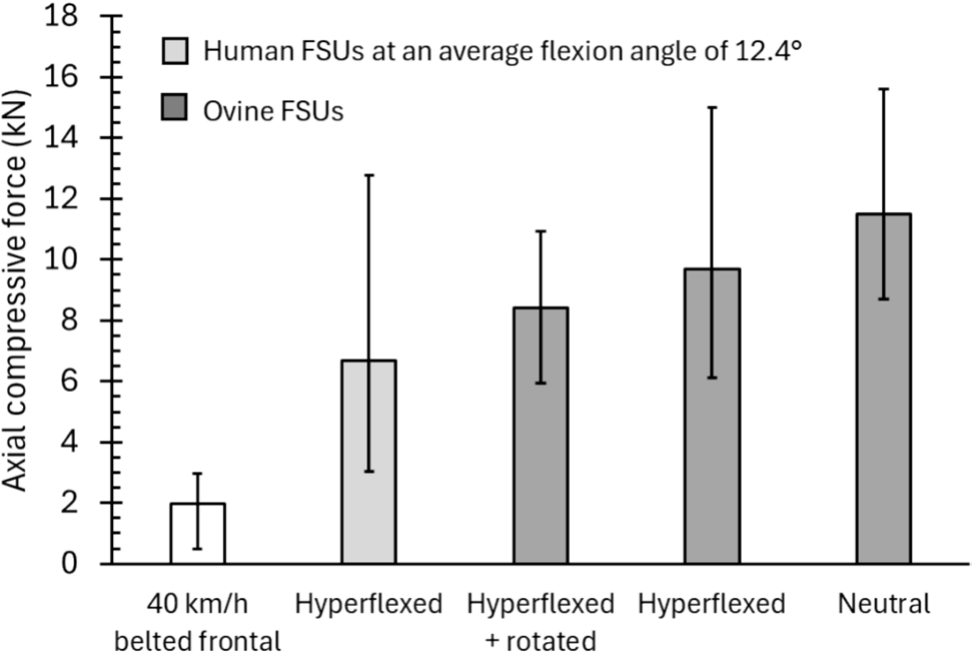
Expected and extreme values of AM50 lower spine compression in a 40-km/h ${\Delta }V$ frontal impact (Eqs. (1) and (2)) compared to the axial compression force measured in 28 hyperflexed human FSUs that sustained isolated disc injury (mass-scaled to AM50) [60, 61] and the axial compression in ovine FSUs that sustained isolated disc injury [62, 63]. Mean and ranges shown for FSU tests

Isolated spinal disc injuries have not been produced experimentally from dynamic loading to an intact human spine [1]. Disc injuries have, on the other hand, been developed in lumbar functional spinal units (FSUs) under specific quasistatic biomechanical loading conditions [60–63]. Adams and Hutton [60, 61] generated nuclear extrusion, annular protrusion, or disc prolapse in 28 samples by compressing hyperflexed lumbar FSUs. Those test samples were not appropriately rehydrated after freezing and long-term storage [64], the loads were applied quasistatically, and the magnitude of the applied hyperflexion was greater than the anatomical range of motion in vivo [1, 65, 66], so the compressive forces required to cause injury in those samples are almost certainly less than would be required in a spine loaded dynamically in vivo. Regardless, even those samples required compressive force exceeding the forces measured in the tests reported here (Figure 4). When mass-scaled to the AM50 for comparison, an average compressive force of 6682 N was applied to those injured FSUs (range 3043 N to 12,786 N). In contrast, the expected value of lower spine compressive force experienced by a belted AM50 in a 40 km/h frontal impact is 1985 N with a maximum upper extreme of 2950 N (Figure 4, left bar). Wade et al. [62, 63] showed that axial compressive force greater than 5930 N was generated in tests that caused disc prolapse in ovine FSUs, even when complex combined flexion and torsional loads were applied (Figure 4, right bars).

Even in impacts as severe as 40 km/h, belted occupants would not be expected to experience cervical spine tension, compression, flexion, or extension exceeding IARVs published by the National Highway Traffic Safety Administration [57, 67]. Kleinberger et al., for example, report neck load tolerance limits for the 5th percentile female of 2080 N in tension, 2520 N in compression, 95 Nm in flexion, and 28 Nm in extension, with the corresponding values for the 50th percentile male of 3300 N, 4000 N, 190 Nm, and 57 Nm [57] (Figure 2a). Neither would belted occupants be expected to experience lower spine compression exceeding the maximum permissible limit (6377 N in the lumbar disc) or, in fact, even the action limit (3434 N in the lumbar disc) defined by the National Institute for Occupational Safety and Health (NIOSH) [58, 59] (Figure 2b).

### Voluntary Activities

The spinal loads generated while performing non-injurious activities have been extensively documented, including those generated while walking, running, skipping rope, tying a shoe, jumping, falling, and engaging in various sports activities [68–80]. Those loads provide important context for the loads generated in the frontal impacts analyzed here. Dynamic loads, such as those generated by sports and other regular activities, including even locomotion, occur over time scales that are relevant to those that occur in low-to-moderate-severity frontal impacts (*viz.*, tens to hundreds of milliseconds) [69–71, 75, 76, 79, 80] and the peak values of those loads can exceed the loads reported here, regardless of ${\Delta }V$. For example, a full golf swing, regardless of skill level, has been reported to generate lumbar spine axial compression force around eight times body weight (BW) [74, 75], while the expected force in a 40-km/h-${\Delta }V$ frontal impact is around 2.6 times BW and the most extreme upper value in any of the tests included here is around 3.8 times BW. Even stooping or squatting generates lumbar spine compression force greater than the expected value in a 40-km/h-${\Delta }V$ frontal impact [68]. A vigorous head shake generates cervical spine flexion moment similar to that expected in a 40-km/h-${\Delta }V$ frontal impact and jumping off a step generates axial compression force similar to the expected 40-km/h-${\Delta }V$ value [70].

**Fig. 5:**
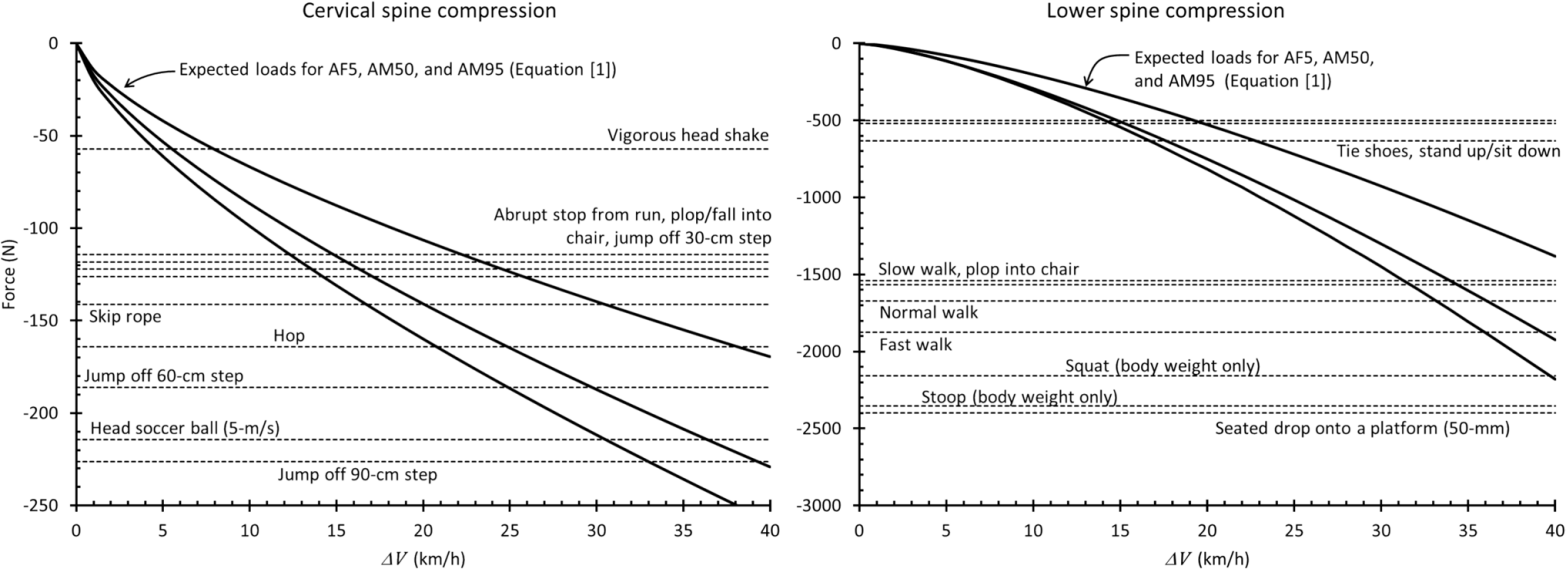
Expected spine compression force as a function of frontal impact ${\Delta }V$ compared to compression force measured during other activities [68–70, 76, 78–80]

Other types of activities generate loads in the range of the loads measured in the frontal impacts considered here (Figure 5). In the cervical spine, for example, the axial compression force was generated while abruptly stopping from a run or plopping into a chair is similar to the expected value at 15-km/h ${\Delta }V$ and hopping or skipping rope is similar to a ${\Delta }V$ of 18-23 km/h. Tying one’s shoes, standing, or sitting generates axial compression force in the lumbar spine similar to the expected value at 15-km/h ${\Delta }V$.

### Disc Injury Mechanism and Tolerance

Acute disc injuries, when objectively diagnosed, typically present with concomitant trauma such as vertebral body fractures and ligamentous injury [1, 14, 81–84]. This clinical observation reflects the well-established biomechanical observation that the intervertebral disc is stronger under dynamic compression and bending than the adjacent bony structures in an intact spine [82, 85–90], even in individuals with degenerated [91] or severely degraded [92] discs. Furthermore, experimental studies have shown that traumatically induced disc herniations, in addition to being accompanied by concomitant trauma, involve extrusion of the nuclear material through fragments of fractured adjacent bone, through the end plate of the disc, or between the endplate and the disc, not through the annulus [60, 82, 84–86, 88, 93]. Thus, there is no known biomechanical mechanism for an acute dynamic load, such as what occurs in a vehicle impact, to cause isolated disc bulges, herniations, or extrusions through the annulus in an intact spine, regardless of the magnitude of loading. The tests analyzed here show that belted occupants in frontal impacts below 40 km/h would not be expected to experience loads of sufficient magnitude to injure discs under any conditions ever studied, including in vitro conditions that unrealistically concentrate loading on poorly preserved discs.

## Discussion

Disc injuries are classified as severity level two or greater on the Abbreviated Injury Scale (AIS) [94, 95]. Belted occupants in low-to-moderate-severity frontal impacts have a very low risk of sustaining a spinal injury exceeding AIS level one (e.g., muscle strains or ligament sprains) [1, 14, 30, 81, 96, 97]. The current study confirms that such occupants experience spine loads below established IARV, below the loads that have caused disc injuries in laboratory testing, and below the loads generated in many activities of daily life.

## Study Limitations

No ATD spine is a perfect biomechanical representation of the human spine, so the distribution of loads throughout the spines of ATDs differs from that in humans. For example, human, THOR, and Hybrid III necks can assume different mode shapes in certain loading conditions [98] and load magnitudes may differ in similar test conditions [32]. Likewise, loads within the human spine are difficult to measure directly and must be calculated from measured kinematics, which is a limitation of the human volunteer and cadaver data reported here. This study is not intended to be a deep exploration of ATD biofidelity in these test conditions, nor of the experimental challenges of determining loads within a human spine, but the data compiled for this paper show that the variability due to test conditions (pulse shape, restraint geometry, etc.) is much greater than any systematic difference in load magnitude between humans and ATDs in anatomical locations for which comparative data are available (compare the cross symbols to the circle symbols in Figure 2) or among different methods of estimating human neck loads. Both human and ATD loads fall within the extreme value functions reported here and the expected value functions optimized to either set of data are similar to the functions optimized to the aggregated data.

Finally, the data reported here are the most extensive set of low-to-moderate-severity frontal impact conditions considered to date in the literature, but no experimental dataset can encompass every possible loading situation that may be encountered in the field. The tests reported here include features that may generate artificially high spinal loads (e.g., universal test bucks and rigidized restraint anchors) and thus the functions reported here may overstate the loads that occur in contemporary vehicles.

## Supplementary Information

Below is the link to the electronic supplementary material.

Supplementary file1 (PDF 1413 KB)

## Data Availability

All data are provided in the Supplement Materials
