# Supplementary material for: Disc Injury and Spine Loads in Low-to-Moderate-Severity Frontal Impacts
Source: Ann Biomed Eng. 2025 Jul 19;53(10):2689–700. doi: 10.1007/s10439-025-03808-w (PMC12457475; doi:10.1007/s10439-025-03808-w)

Raw data and test information

Scaled and unscaled experimental data with expected (solid bold line), upper extreme, and lower extreme (dashed lines) spinal loads expressed as functions of change in velocity in frontal impacts (see Equations [1] and [2] and Tables 1 and 2).


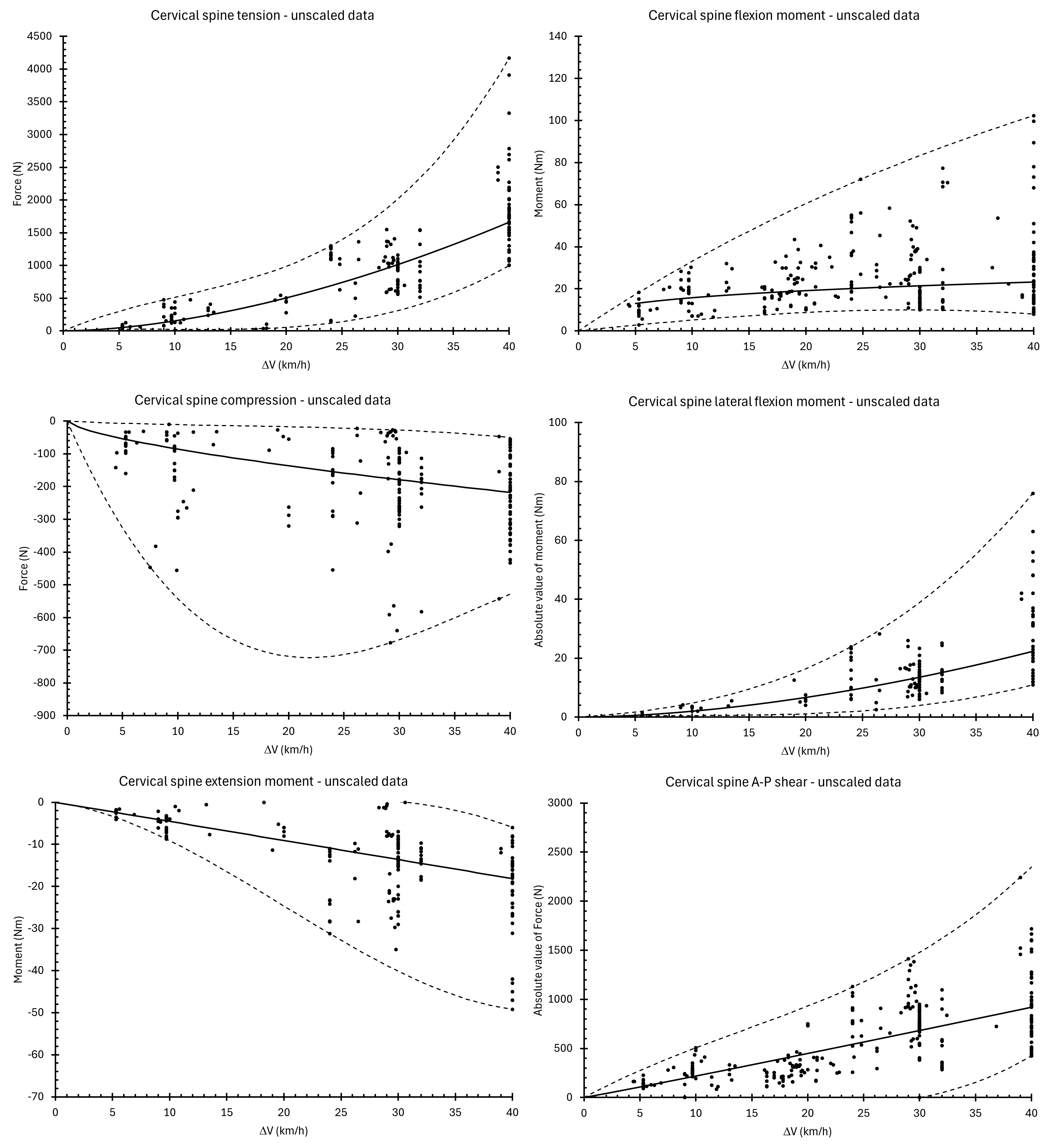

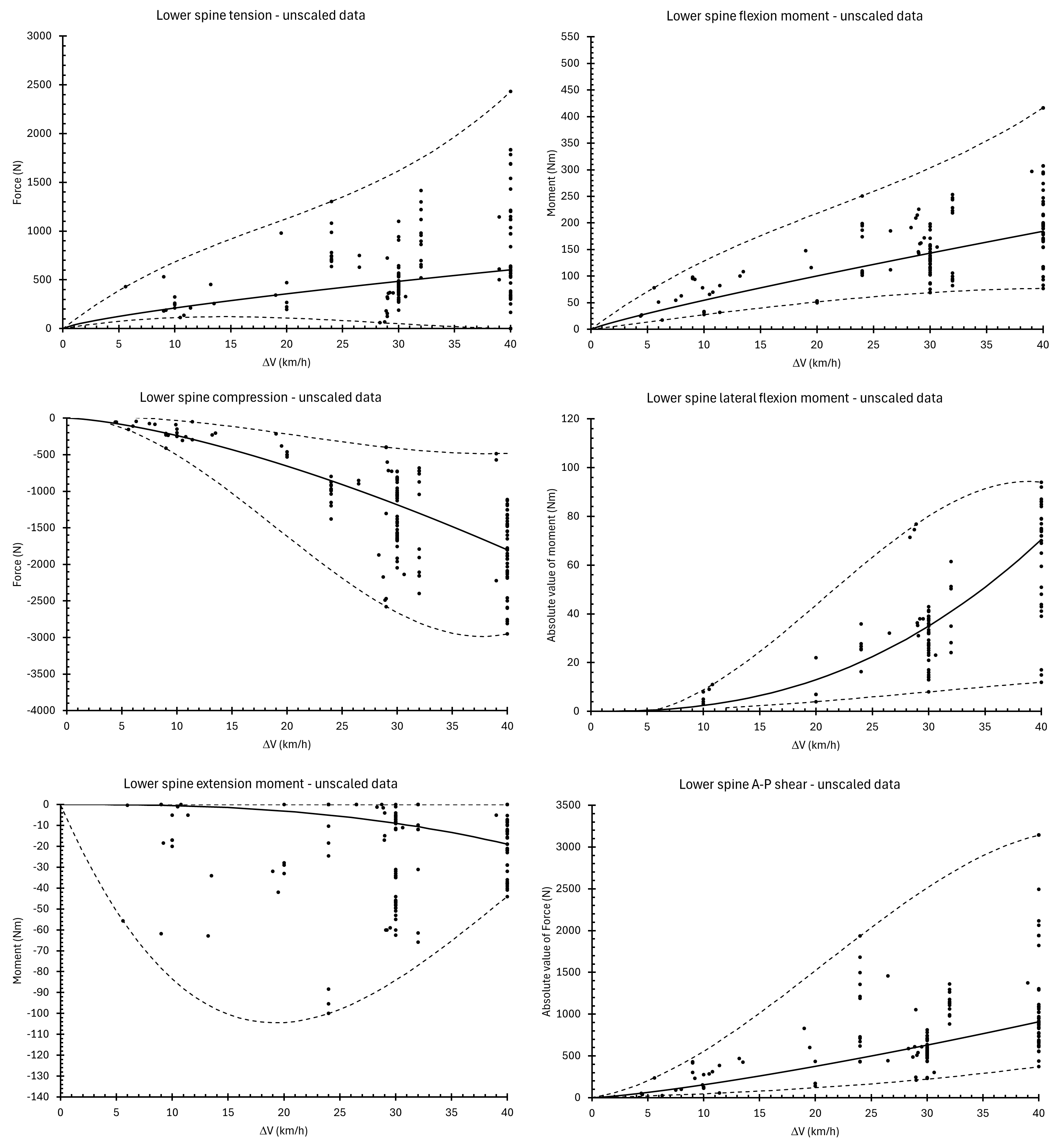

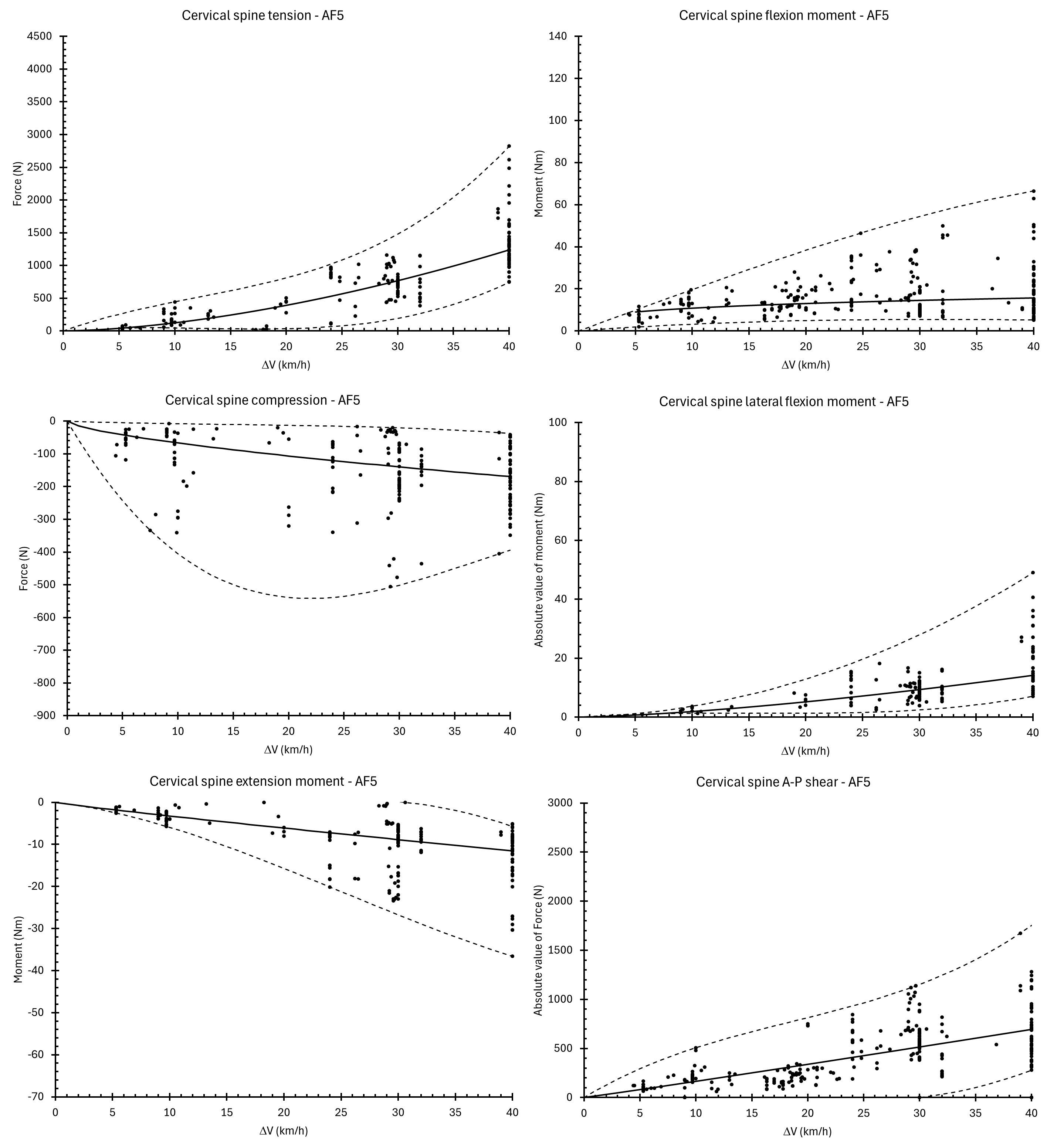

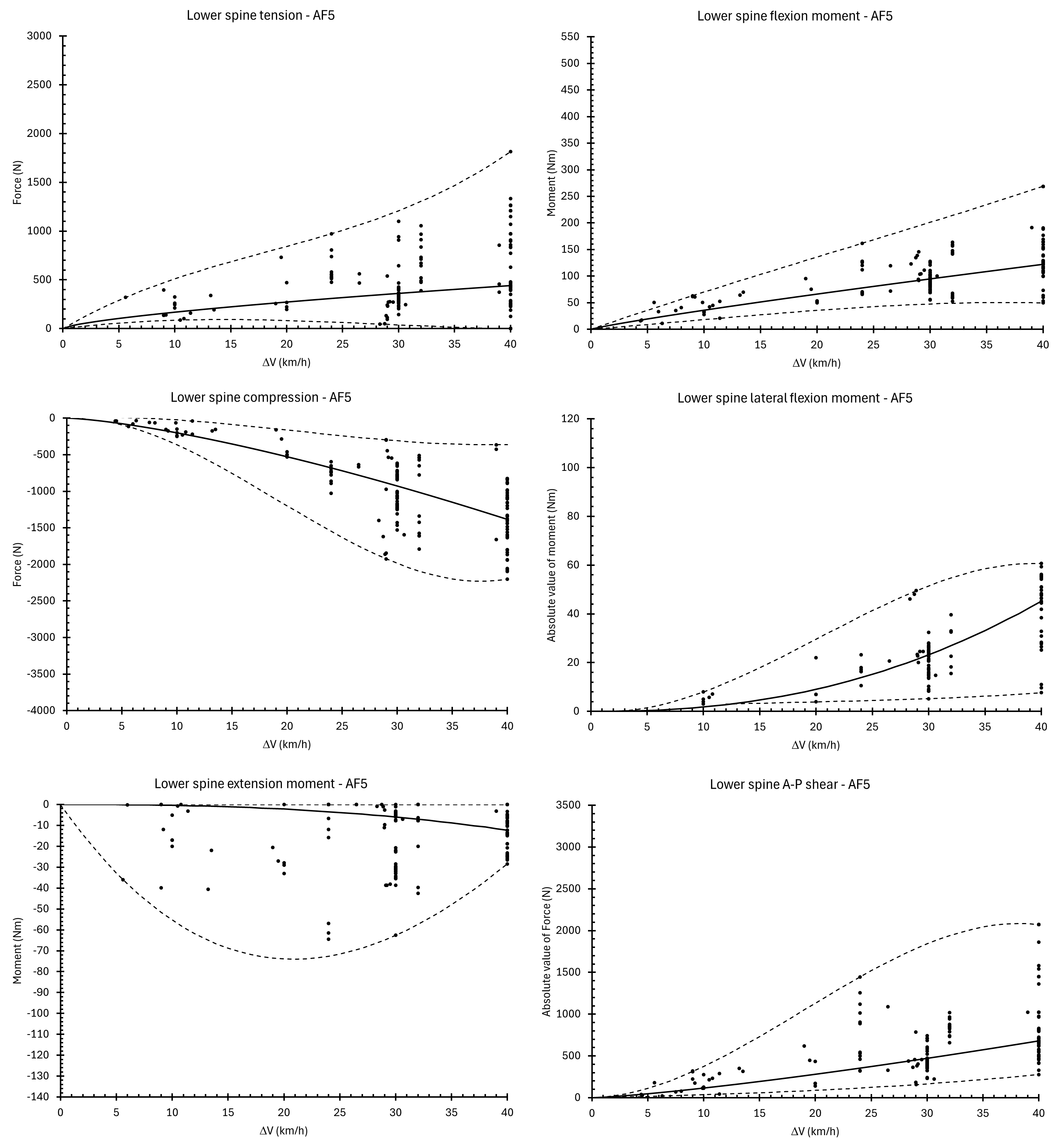

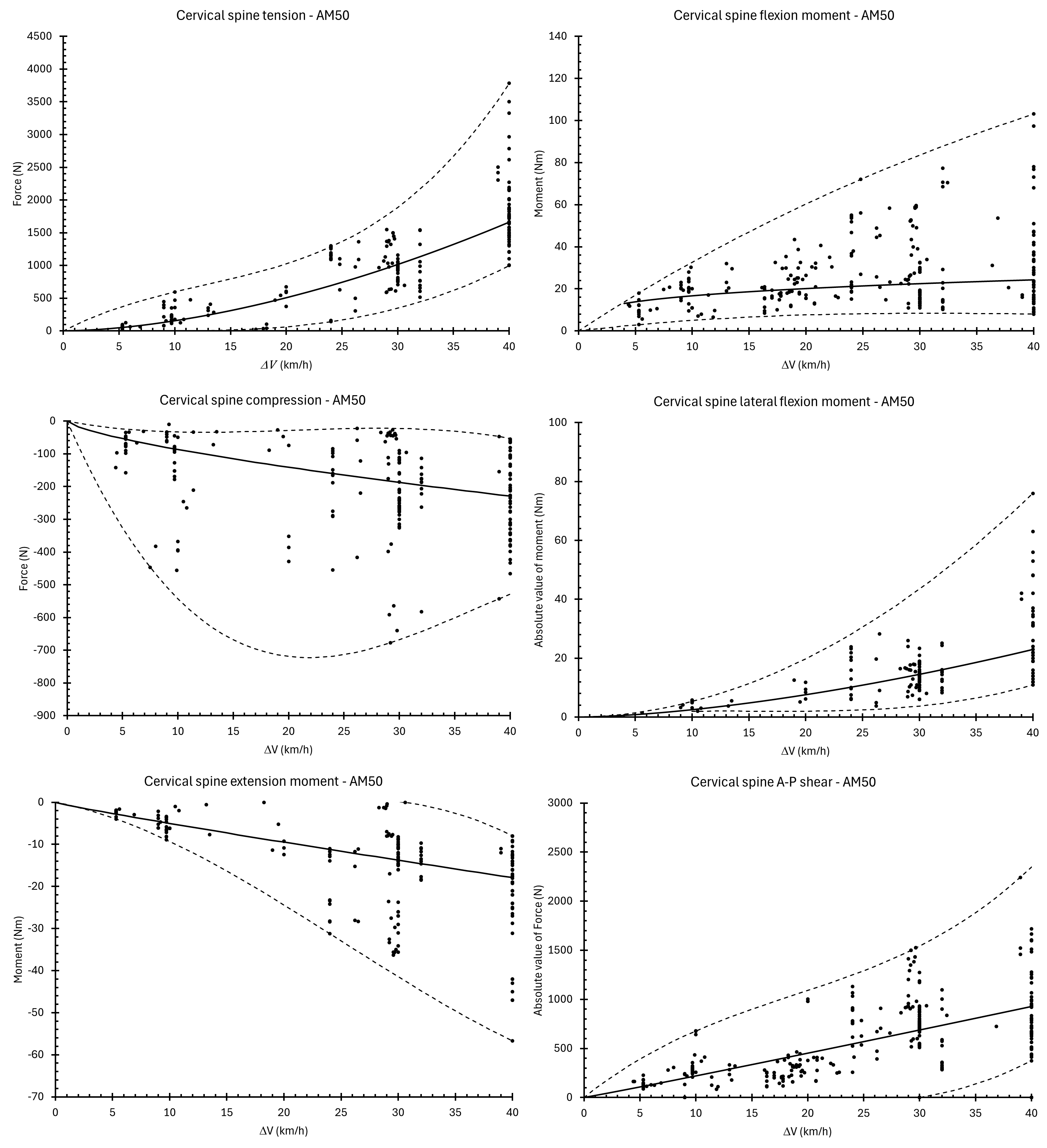

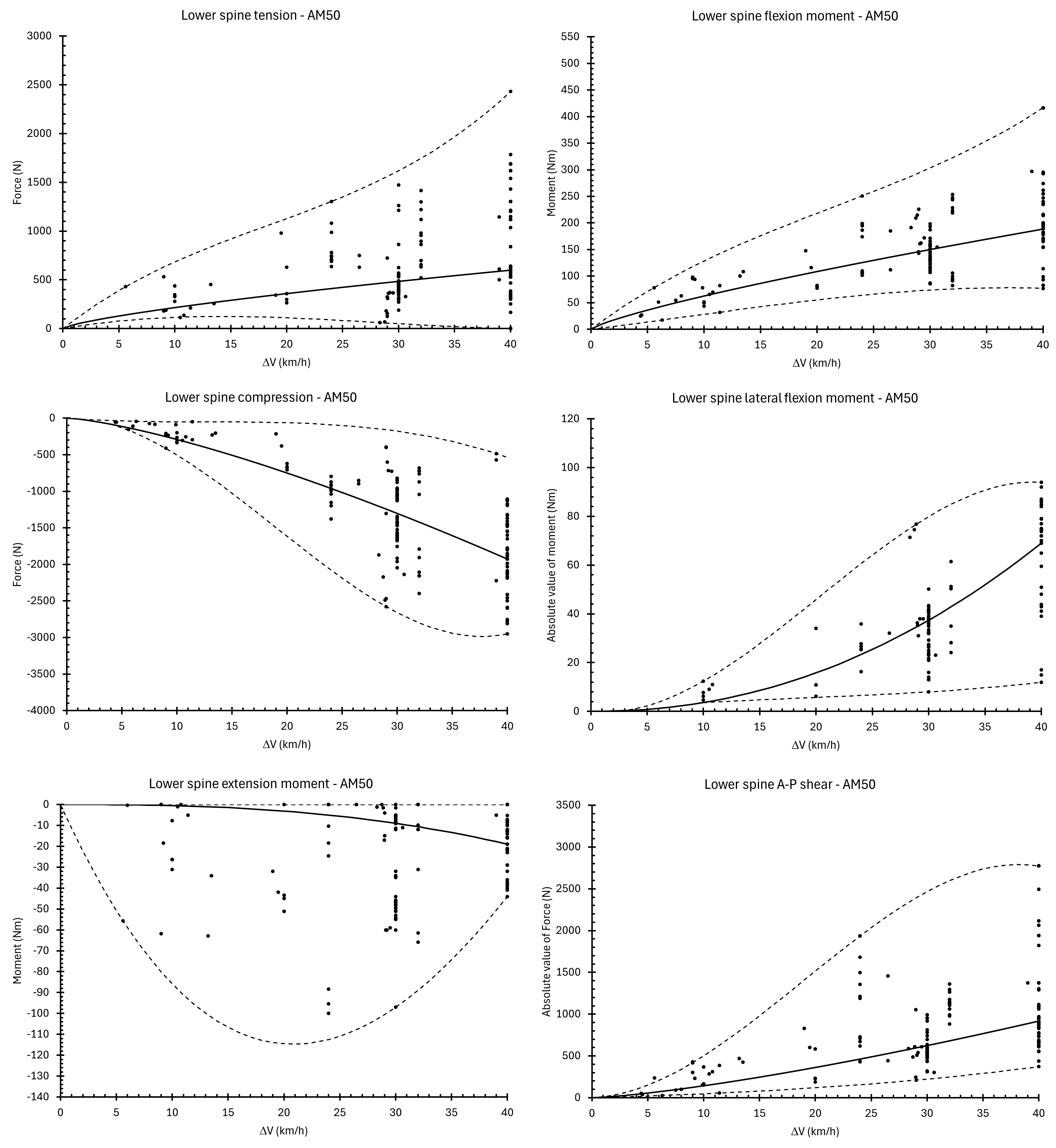

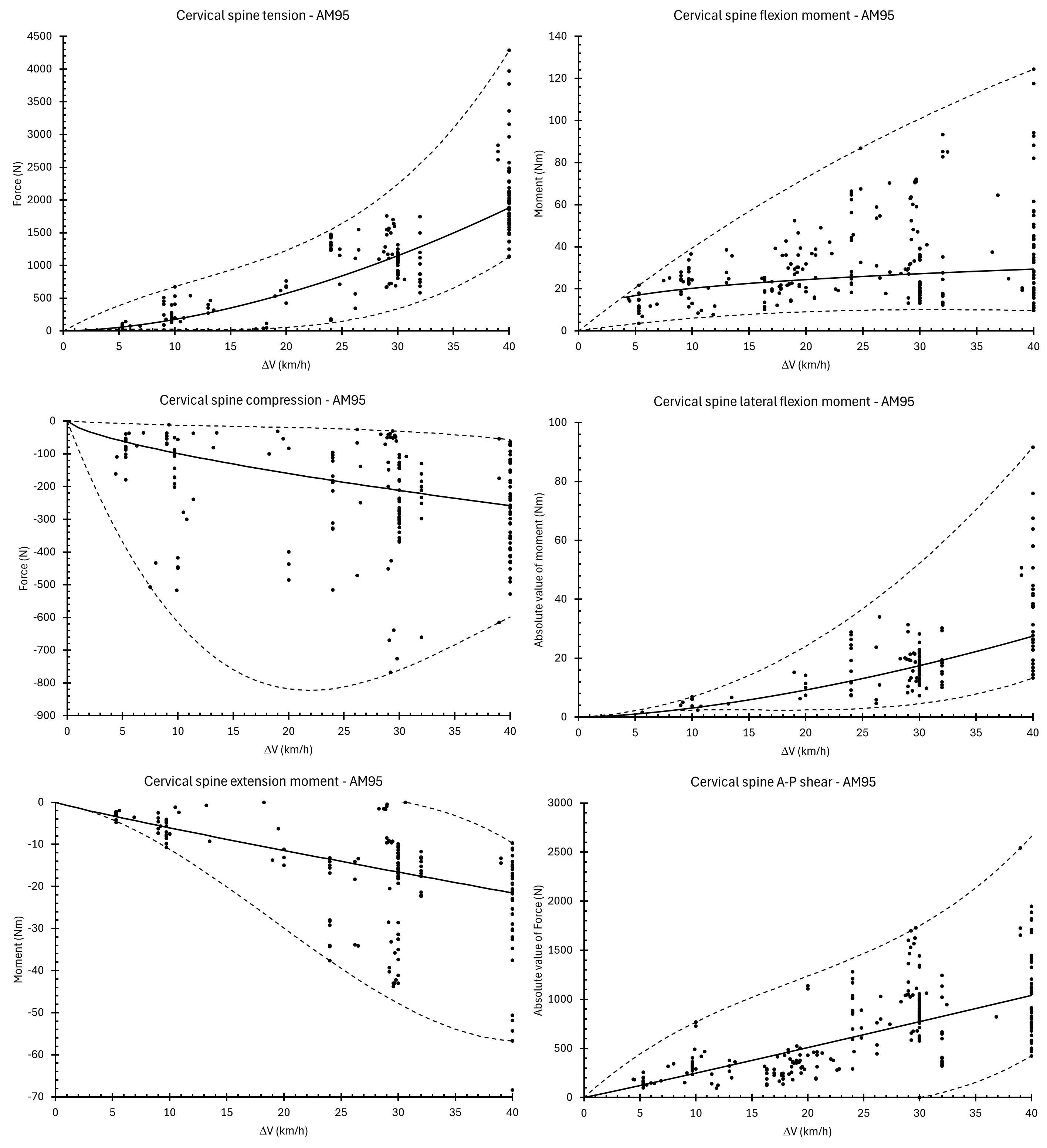

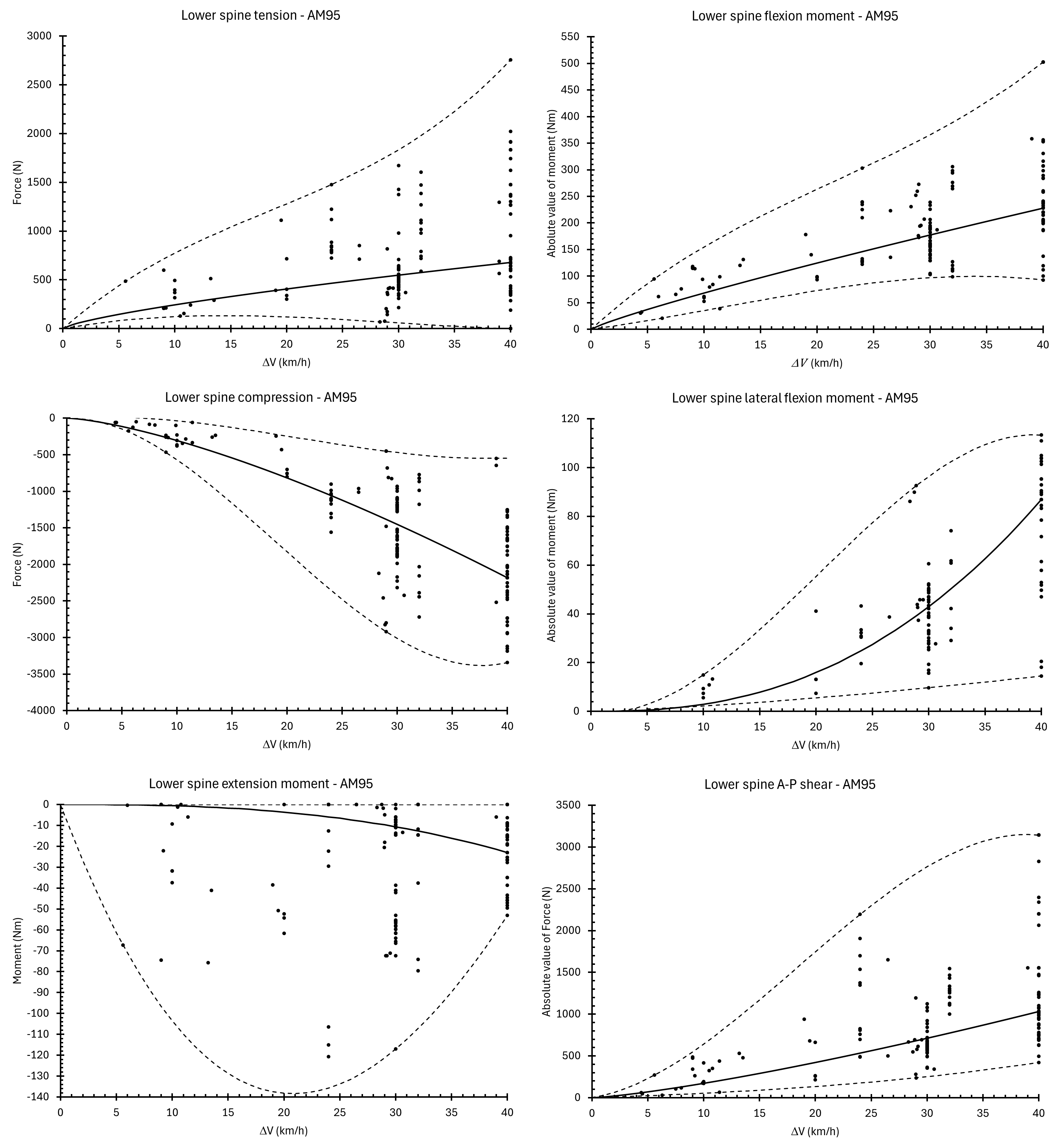

Supplement: Supplementary file 1 — Supplementary file1 (PDF 1413 KB) [file 10439_2025_3808_MOESM1_ESM.docx]
